# Different care mode alter composition and function of gut microbiota in cerebral palsy children

**DOI:** 10.3389/fped.2024.1440190

**Published:** 2024-08-22

**Authors:** Jinli Lyu, Xiaowei Zhang, Shenghua Xiong, Hui Wu, Jing Han, Yongjie Xie, Feifeng Qiu, Zhenyu Yang, Congfu Huang

**Affiliations:** ^1^ Department of Obstetrics and Gynecology, Peking University Shenzhen Hospital, Shenzhen, China; ^2^ Department of Pediatrics, Longgang District Maternity and Child Healthcare Hospital, Shenzhen, China; ^3^ Department of Pediatrics, Hexian Memorial Affiliated Hospital of Southern Medical University, Guangzhou, China; ^4^ Department of Critical Medicine, The First Affiliated Hospital, Jinan University, Guangzhou, China; ^5^ Department of Microbial Research, WeHealthGene Institute, Joint Laboratory of Micro-Ecology and Children’s Health, Shenzhen Children’s Hospital, Shenzhen WeHealthGene Co., Ltd., Shenzhen, China

**Keywords:** cerebral palsy, Gut microbiota, family-centered care mode, welfare-centered care mode, 16S rRNA sequence

## Abstract

**Introduction:**

Specialized care is essential for the recovery of children with cerebral palsy (CP). This study investigates how different care modes impact the gut microbiota.

**Methods:**

Fecal samples from 32 children were collected, among whom those cared for by family (*n* = 21) were selected as the observation group, and those cared for by children's welfare institutions (*n* = 11) were selected as the control group (registration number of LGFYYXLL-024). The gut microbiota profiles were analyzed.

**Results:**

There was no significant difference in the α-diversity of the gut microbiota and the abundance at the phylum level. However, at the genus level, the observation group showed a significant increase in the abundance of butyrate-producing bacteria *Bacteroides* and *Lachnospiracea incertae sedis* (*P* < 0.05), and a significant decrease in the abundance of opportunistic pathogens *Prevotella*, *Clostridium* cluster IV, *Oscillibacter*, and *Fusobacterium* (*P* < 0.05). Additionally, lipid metabolism, carbohydrate metabolism, transcription, cellular processes and signaling, and membrane transport were significantly upregulated in the observation group. Lipid metabolism was positively correlated with *Bacteroides* and *Lachnospiracea incertae sedis*, indicating a positive impact of the family-centered care mode on bacterial metabolism processes.

**Discussion:**

This study highlights that the family-centered care mode had a positive impact on the composition and function of the gut microbiota. The study provides valuable insights into the relationship between care mode and gut microbiota, which can inspire the development of interventions for cerebral palsy.

## Introduction

Cerebral palsy is a non-progressive central physical disability of childhood resulting from brain damage in the developing fetus and/or infant (1). Additionally, cerebral palsy has been associated with gastrointestinal (GI) symptoms such as dysphagia, gastroesophageal reflux, feeding difficulties and constipation, with a reported prevalence of 80%–90% (2). There is an obvious correlation between cerebral palsy and an imbalance of gut microbiota. The microbiota-gut-brain axis is a bidirectional pathway that connects the enteric nervous system (ENS) and central nervous system (CNS) via neural-endocrine-immune system (3). Neuro/immune-active substances from the intestinal lumen can penetrate the gut mucosa, be transported by blood cross the blood- brain -barrier (BBB) and affect CNS (4). Additionally, gut microbiota influence brain chemistry and development (5). Conversely, the CNS affects intestinal permeability by activating hypothalamic-pituitary-adrenal (HPA) axis, and it regulates the gut microbiota composition directly or through nutrient availability via neuropeptides released from sensory nerve fibers (4). A previous study found differences in the composition and diversity of gut microbiota between children with cerebral palsy and healthy children (6). The changes in gut microbiota were closely related to digestive dysfunction, brain development and immune function in children with cerebral palsy (7).

Dietary structure can affect the digestive function and nutrient condition of children via the changes of gut microbiota. It has been demonstrated that children with cerebral palsy on a liquid diet had constipation and varying degree of malnutrition. Moreover, symbiotic pathogenic bacteria such as *Collinsella, Alistipes, Eggerthella* were rich in children on a liquid diet, while butyric acid-producing and lactic acid-producing genera like *Lachnoclostridium, Dorea, Ruminococcus, Faecalibacterium, Roseburia, Lactobacillus* were enriched in children on a general diet, demonstrating a significant correlation between changes in the above genera and constipation and malnutrition (8).

Apart from diet structure affecting the gut microbiota, living environment, disease and antibiotics have also attracted too much attention (9). The family-centered care mode is considered a best practice not only for children with cerebral palsy but also for parents (10). Mothers of children with cerebral palsy experienced decreased psychiatric symptoms under the family-centered care mode (11). In another study, reduced anxiety occurred in parents under family-centered services which offered knowledge and skill development, support, opportunities to voice concerns (12). Besides that, skin contact between family member and children, as well as dietary diversity in family, can promote the growth of beneficial genera such as *lactobacillus* and reduce the incidence of GI disorders (9, 13). Microbial exchange of family members is facilitated by leaving microbes from their bodies on the surfaces they touch, resulting in similarities in the composition of gut microbiota (14–16). For example, rural children showed a higher abundance of *Bacteroidetes* compared to urban children, while *Firmicutes* were abundant in urban children (17). There is a markedly increased tendency towards the diversity of microbiota in children living on farms compared to rural children, which is related to exposure to the microbiot from barns and animal sheds (18). These studied indicated differences in gut microbiota of children based on the living environment. In this study, children with cerebral palsy were divided into a family-centered care mode group and a welfare-centered care mode group to investigate the effect of different care modes on the composition and function of gut microbiota.

## Methods

### Patients recruitment

This study recruited a total of 32 children diagnosed with cerebral palsy by Shenzhen Longgang District Maternity & Child Healthcare Hospital, Guangdong, China. Among these children, 21 who received family-centered care were selected as the observation group, while 11 who were cared for by Children Welfare were selected as the control group. All children met the diagnostic criteria for cerebral palsy and did not have liver disease, chronic GI dysfunction and recent recovery from GI infection, or genetic metabolic diseases. Children who had used antibiotics or probiotics within the past two weeks were also excluded from the study.

### Sample collection and 16s rRNA sequencing

Approximately 5 g of feces were collected from the children in both groups and stored at −80°C within 1 h after collection. Bacterial DNA were extracted from fecal samples using PowerSoil® DNA Isolation Kit (MoBio, America), followed by amplification of V3-V4 region of 16S rRNA and sequencing. Sequencing was executed using the Illumina Miseq platform.

### Data processing and statistical analysis

Clean data was obtained after filtering out low-quality data and then sequencing splicing was performed using FLASH software (v1.2.11). The spliced sequences were used to analyze OTU clusters and species classification via USEARCH and RDP classifier. The abundances of bacteria at the phylum and genus level were calculated for further comparison between two groups.

Principle components analysis (PCA) was performed by ade4 package in R (v3.3.3) based on the relative abundance of all samples in genus level. Wilcoxon test were used to compare the two groups at phylum and genus level. *P* < 0.05 were selected for statistical significance. PICRUSt (phylogenetic investigation of communities by reconstruction of unobserved states) was applied to generate the functional content of the microbiota based on 16S rRNA OTUs profiling. KEGG pathway predicitions were then made based on the abundance of KEGG Orthology (KO) calculations. Clinical data from two groups were statistically analyzed using SPSS (v22.0). Age, weight, and height were calculated and displayed with mean ± SD. The Chi-square test and independent sample *t*-test were used for statistical analysis.

## Results

### Participants characteristics and data output

A total of 32 children with cerebral palsy aged between 3 and 10 were recruited for this study. The average age of observation groups were 5.87 ± 0.52 and control group were6.58 ± 0.45, with no significant difference (Table 1). Moreover, there were no statistically significant differences between the two groups in the other parameters, including sex, weight, and height. It is evident that the above factors had no significant impact on the results of this study.

**Table 1:** Participants characteristics between two groups.

Parameters	Observation group (*n* = 21)	Control group (*n* = 11)	*P-*value
Age (years old)	5.87 ± 0.52	6.58 ± 0.45	0.264
Gender (boys/girls, ratio)	12/9 (1.3)	6/5 (1.2)	0.714
Weight (kg)	16.37 ± 1.15	16.40 ± 1.84	0.425
Height (cm)	98.25 ± 1.21	93.52 ± 1.42	0.084

### Differences between two groups in the structure of gut microbiota

**Figure 1:**
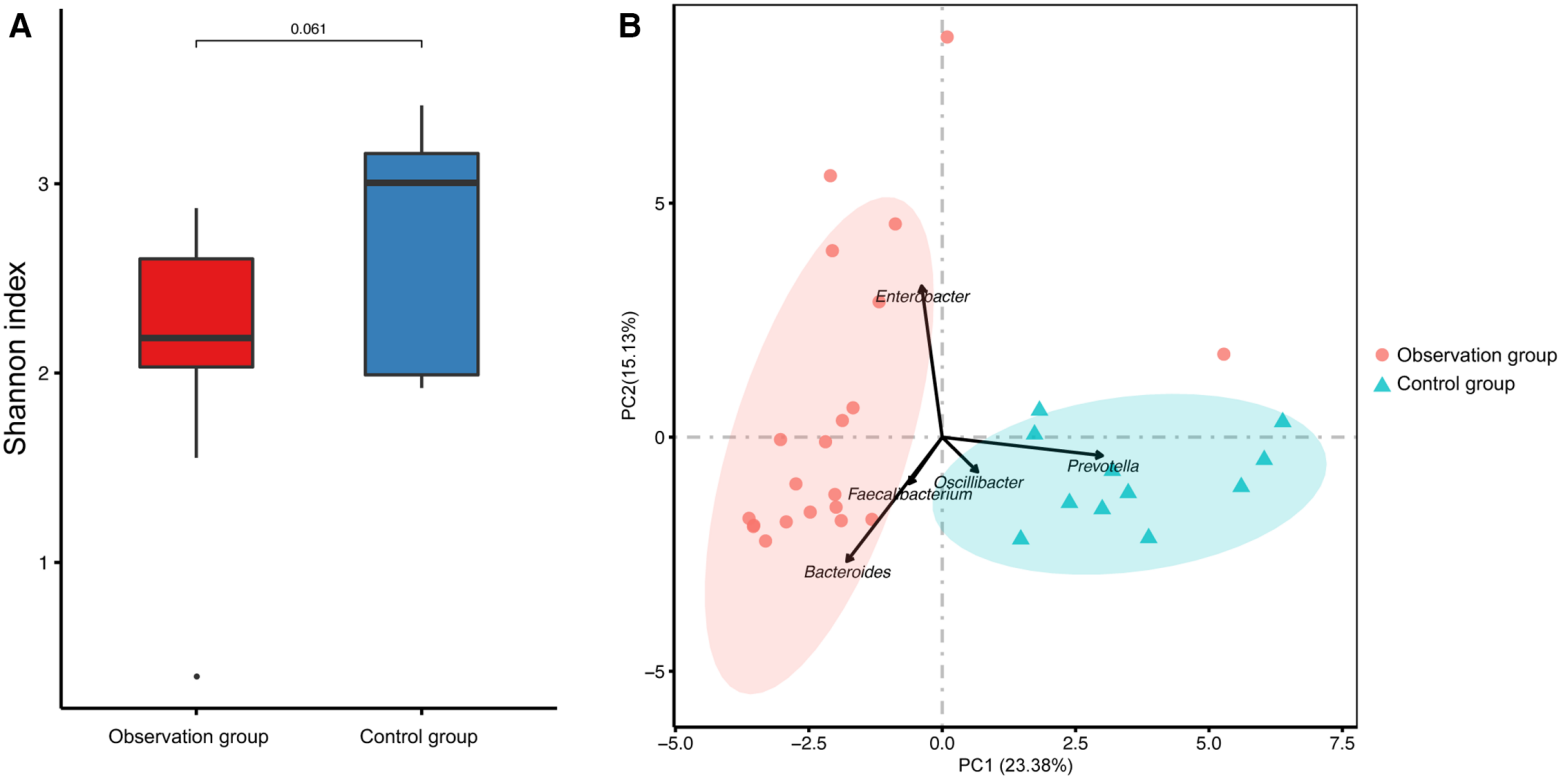
The microbial diversity of fecal samples. (**A**) The a-diversity of fecal microbiota presented by Shannon- Winner index. (**B**) The principal component analysis (PCA) of the two groups were presented.

The richness of the gut microbiota was assessed using the α-diversity, which was calculated using the Shannon-Weiner index based on OTUs. The Shannon-Weiner index was decreased in the observation group compared to the control group, without statistically difference (Figure 1A). PCA plots were generated based on Bray-Curtis distance to assess the similarity of the gut microbiota between the two groups. The samples from the two groups clustered separately (Figure 1B), with *Bacteroides*, *Faecalibacterium*, and *Enterobacter* contributing to observation group, and *Oscillibacter* and *Prevotella* contributing to the control group.

### Differences of the gut microbial composition between two groups

**Figure 2:**
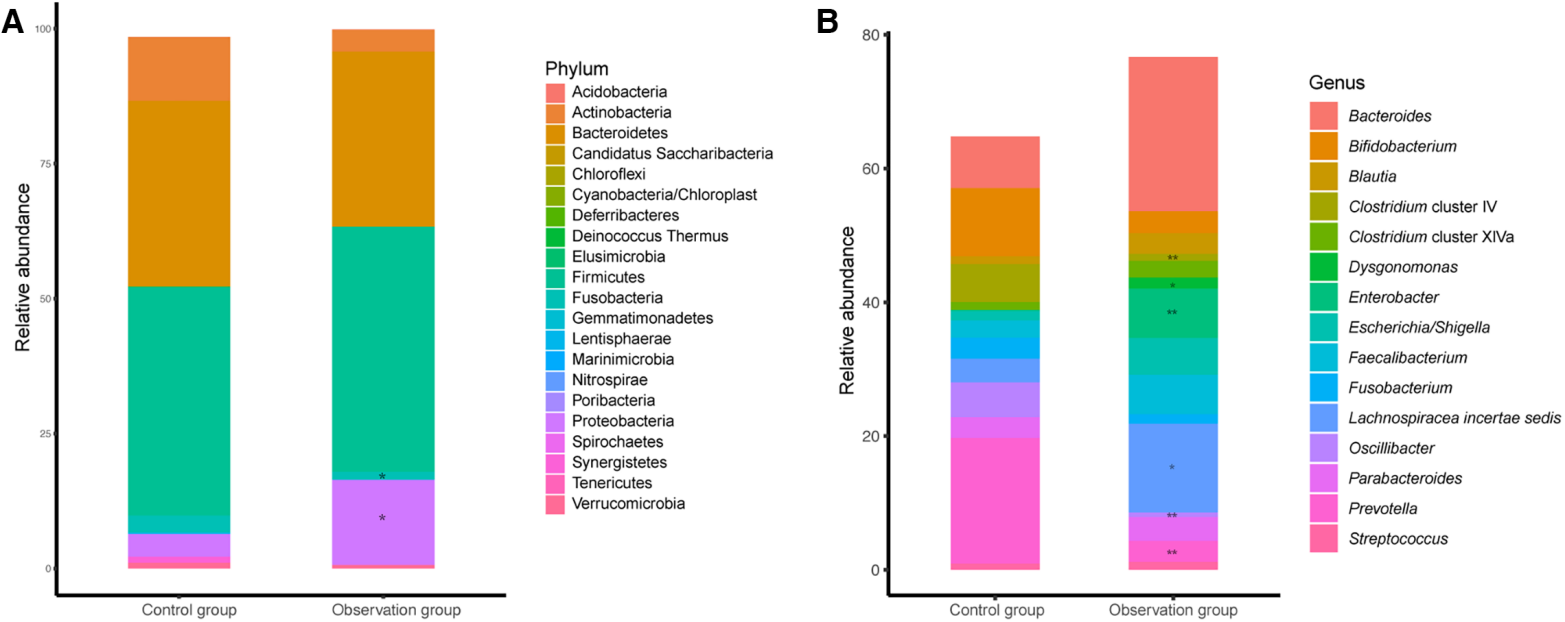
The microbial taxonomy of fecal microbiota at phylum (**A**) and genus (**B**) level. Remarks: *indicates *P* < 0.05, **indicates *P* < 0.01, ***indicates *P* < 0.001, statistically significant differences between the two groups. The higher the number of asterisks, more significant the difference.

The relative abundance of each species was calculated to investigate the influence of different care mode on the composition of gut microbiota at the phylum and genus levels, including a total of 21 phyla and top 15 abundant genera (Figure 2). The dominate phylum observed in both groups were Bacteroidetes and Firmicutes, with no statistical difference between the two groups. Firmicutes accounted for 45.41% and 42.30% in observation and control groups, repectively, while Bacteroidetes accounted for 32.43% and 34.43% in observation and control groups, repectively. The abundance of Proteobacteria was significantly higher in observation group compared to control group, while the abundance of Fusobacteria was lower in observation group than in observation group (Figure 2A). At the genus level, the abundance of *Lachnospiracea incertae sedis, Enterobacter, Streptococcus* and *Dysgonomonas* were significantly higher in observation group than in control group, while *Prevotella*, *Clostridium* cluster IV and *Oscillibacter* were significantly lower in observation group than in control group (Figure 2B).

### Comparison of metabolism pathways of gut microbiota between two groups

**Figure 3:**
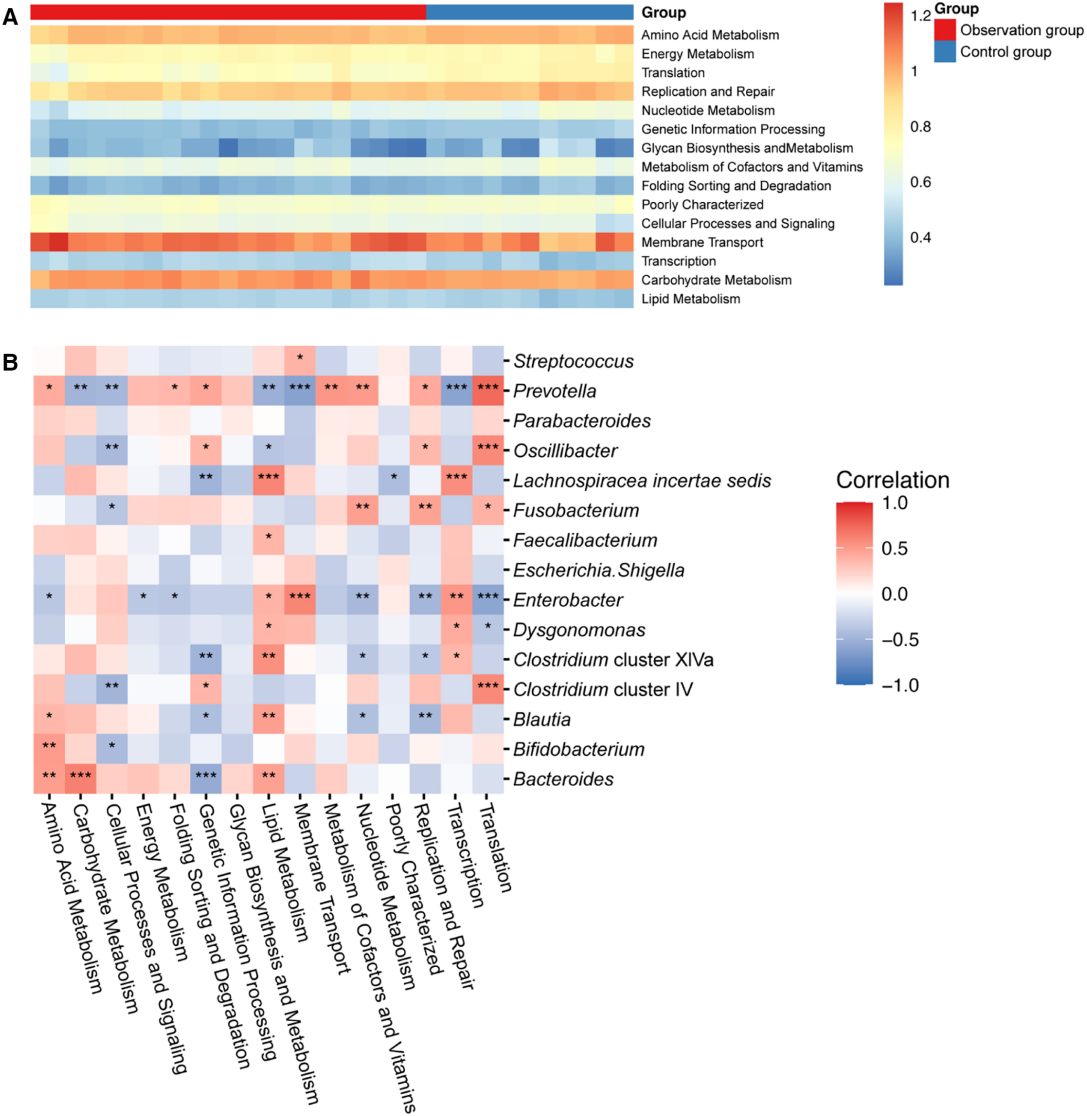
(**A**) Heat map showing the predicted KEGG pathways differing in the gut microbiota of two groups. (**B**) Correlation analysis revealed the relationship between KEGG pathway and gut microbiota. Remarks: *indicates *P* < 0.05, **indicates *P* < 0.01, ***indicates *P* < 0.001, ****indicates *P* < 0.0001, statistically significant differences between gut microbiota function and bacterial genera. The higher the number of asterisks, the more pronounced the correlation.

We further idenfied the changes in gut microbial function and metabolic activity between the two groups of children. Out of a total of 37 annotated KEGG pathway, 16 were selected based on their relative abundance (Figure 3A). Compared to the control group, lipid metabolism (*P* < 0.001), carbohydrate metabolism (*P *< 0.01), transcription (*P *< 0.01), cellular processes and signaling (*P *< 0.05), and membrane transport (*P* < 0.05) were significantly upregulated in the observation group.

To investigate the relationship between gut microbiota and metabolism pathways, we utilized correlation analysis to explore the correlation between the top 15 abundant genera and 16 significant KEGG pathway using Spearman's correlation coefficient. As shown in Figure 3B, *Bacteroides* was positively correlated with metabolism of nutrients, like amino acid metabolism, carbohydrate metabolism and lipid metabolism, while negatively correlated with genetic information processing. Moreover, *Enterobacter, Dysgonomonas* and *Lachnospiracea incertae sedis* was positively correlated with lipid metabolism. However, *Prevotella, Oscillibacter* and *Clostridium* cluster IV showed a contrary correlation. They displayed a positive correlation with amino acid, nucleotide, cofactors and vitamins metabolisms, but a negative correlation with carbohydrate and lipid metabolism.

## Discussion

This study compared the gut microbiota composition of children with cerebral palsy under family-centered care mode and welfare-centered care mode. We found that while there was no significant difference in *α*-diversity between the two groups, the gut microbial composition were different. PCA also verified that the structure of the gut microbiota was significantly different between the two groups. Although the abundance of Bacteroidetes was similar in both groups, the dominated genus was different. The observation group was dominated by *Bacteroides*, while *Prevotella* was dominant in the control group. *Bacteroides* is a prevalent gut genus associated with the degradation of plant polysaccharides, which cannot be digested by the human body. *Bacteroides* also contribute to the metabolism of high fat and proteins, providing 10%–15% of the energy from food for the human body (19–21). Previous studies found that *Bacteroides* level was decreased in patients with mental illnesses such as major depressive disorder (MDD) (22), Autism spectrum disorder (ASD) (23), dementia (24). Therefore, the enrichment of *Bacteroides* in observation group may be due to a more balanced and diversified diet under the family-centered care mode, which is more favorable for the recovery of children with cerebral palsy. However, *Prevotella* may play a role in the development of inflammation response and may further be associated with mental illness throuth the brain-gut axis (25). *Prevotella* was considered as the primary microbiota predictor for caries due to its high activity in producing carbohydrate-derived acid (26). Besides, *Prevotella* was enriched in the gut of children with cerebral palsy according to the previous study (7). The persistance of *Prevotella* in the gut of children with cerebral palsy continually induces gut dysbiosis leading to increased mucosal permeability (27) of the gut-brain axis (28). Increased permeability can lead to elevated serum endotoxin levels, which activate the immune system and promote IL-1β production (29). Therefore, the higher abundance of *Prevotella* in the control group may hinder the recovery of children with cerebral palsy under the welfare-centerd care mode. Similarily, the genera of Firmicutes in the two groups were also different. *Lachnospiracea incertae sedis* was enriched in the observation group, while *Oscillibacter* was enriched in the control group. *Lachnospiracea incertae sedis* is an important butyrate-producing bacteria in the gut, which helps maintain the homeostasis of the gut microenvironment. It can decompose carbohydrates into short-chain fatty acids (SCFAs) and promote protein synthesis (30, 31). SCFAs influence immune cells and immune modulators to maintain homeostasis (32, 33). High abundance of *Oscillibacter*, a gram-negative bacterium, is associated with chronic intestinal inflammation and depression (34, 35). Gram-negative bacteria have lipopolysaccharides (LPS) in their outer cell membrane, which interact with macrophages and stimulate the immune response by releasing pro-inflammatory cytokines (36). The strains enriched in the observation group appear to be more beneficial for the recovery of children with cerebral palsy, while the strains in the control group may cause inflammatory reactions. Additionally, *Fusobacterium* were found to be higher in the control group than in the observation group. *Fusobacterium* is a normal oral bacterium, and its presence in the intestine can inhibit immune response and promote the transformation of inflammation into malignancy, which is closely associated with colorectal cancer (37). This suggests that oral care should be strengthened for children in the control group to prevent the entry of oral pathogens into the intestine and further caused the gut microbiota disorder. Notably, *Dysgonomonas* was only detected in the observation group, which is primarily involved in the decomposition of lignocellulose and providing nutrients to the host.

The two groups of children differed in their gut microbiota composition, leading to corresponding differences in their gut microbiota functions. Specifically, there were significant differences in lipid metabolism. Our result also indicate that *Bacteroides* and *Lachnospiracea incertae sedis* were positively correlated with lipid metabolism, while *Prevotella*, *Oscillibacter* and *Fusobacterium* were negatively correlated with lipid metabolism. Lipid metabolism are known to affect host metabolism and immune activity through their metabolites (38). Besides, *Bacteroides* and *Lachnospiracea incertae sedis* increases the production of SCFAs in observation group. Therefore, these significaltly changed genera may affect the recovery of children with cerebral palsy by influencing the lipid metabolism in the gut microbiota.

However, the study also has some limitations. It is lack of many cofactors which can affect the composition of gut microbiota, including specific analysis of dietary components (Supplementary Material Figure S1), detection of microorganisms in the living environment, and investigation of the use of antibiotics and probiotics. Although, it is hard to calculate the sample size due to limited published reports, the sample size in this study is relatively small. Therefore, we will expand the sample size or conduct multi-center studies to gain a broader understanding of the factors that affect the gut microbiota of children with cerebral palsy. PICRUSt2 relies on reference genomes to predict metagenomic functional content, which can lead to inaccuracies, particularly for novel or underrepresented taxa. This limitation can affect the reliability of functional predictions. To address these limitations, we intend to incorporate metabolomics in future studies.

## Conclusion

There are many factors that affect the gut microbiota of children with cerebral palsy, and our previous research suggested that diet structure was one of the most important factors. The findings of the this study suggested that family-centered care mode had a positive impact on the composition and function of the gut microbiota of children. The study provides valuable insights into the relationship between care mode and gut microbiota, which can inspire the development of interventions to cerebral palsy.

## Data Availability

The datasets presented in this study can be found in online repositories. The names of the repository/repositories and accession number(s) can be found below: https://www.ncbi.nlm.nih.gov/, PRJNA968147.

## Supplementary material

Supplementary Figure S1 The percentage of children consuming different ranges of food types daily. The number marked on the top of the bar denote the case number. We referred to the Dietary Reference Intakes for China (2022 edition) to define the intake levels for each type of food. The intake within the recommended amount is classified as Family/Welfare-normal group, intake levels below the recommendation are categorized as Family/Welfare-low group, and levels above the recommendation are categorized as Family/Welfare-high group.
