# Supplementary figures and images for: Different care mode alter composition and function of gut microbiota in cerebral palsy children
Source: Front Pediatr. 2024 Aug 22;12:1440190. doi: 10.3389/fped.2024.1440190 (PMC11374594; doi:10.3389/fped.2024.1440190)

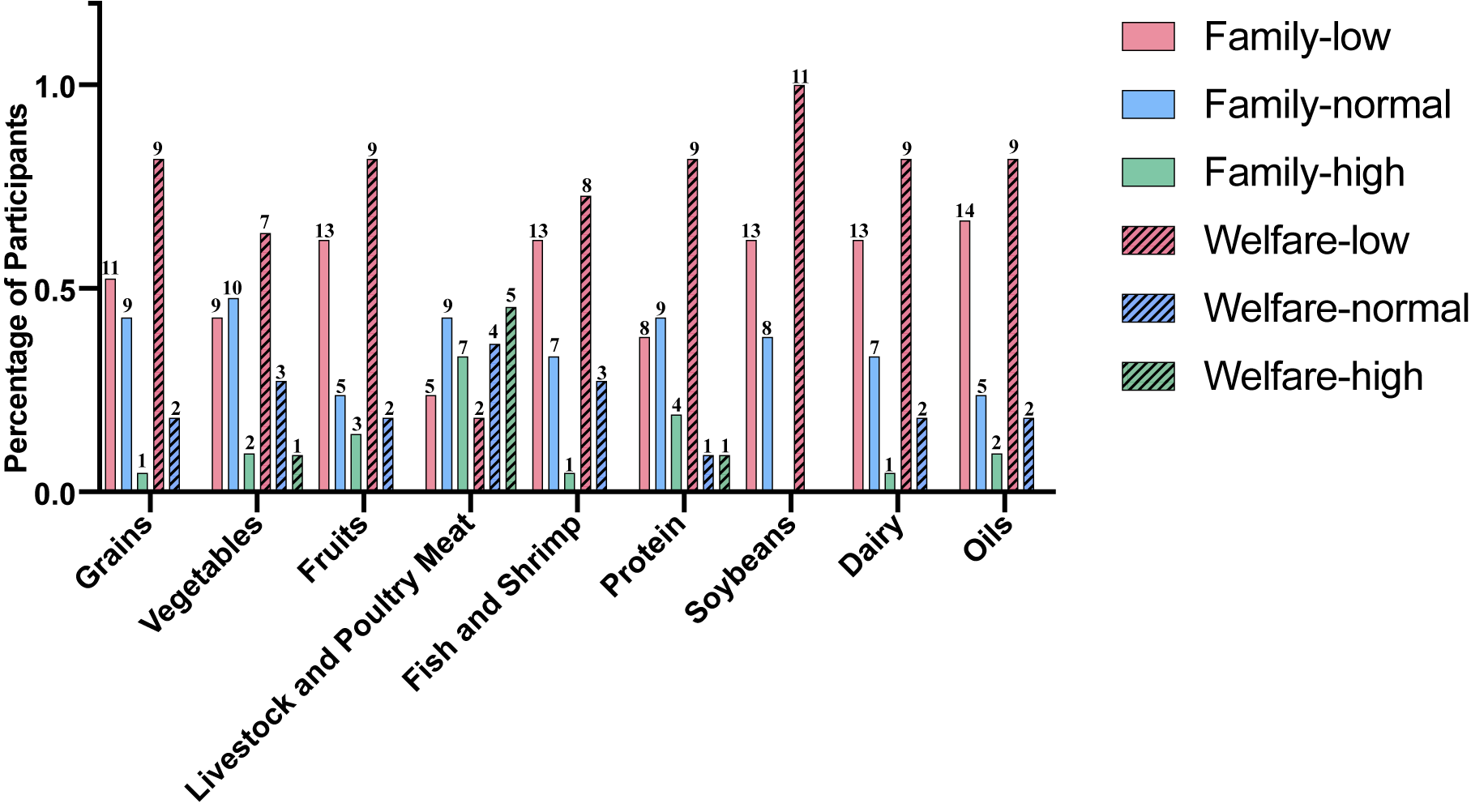

Supplement: Supplementary Figure S1 [file Image1.tif]
